# Supplementary material for: Hybrid Lead-Halide Polyelectrolytes as Interfacial Electron Extraction Layers in Inverted Organic Solar Cells
Source: Polymers (Basel). 2020 Mar 27;12(4):743. doi: 10.3390/polym12040743 (PMC7240626; doi:10.3390/polym12040743)
Supplement: Supplementary file 1 [file polymers-12-00743-s001.pdf]

Supporting Information for:

Hybrid lead-halide polyelectrolytes as interfacial electron extraction layers in inverted organic solar cells

Jin Hee Lee [1], Yu Jung Park [1], Bright Walker [2,*] and Jung Hwa Seo [1,*]

[1] Department of Materials Physics, Dong-A University, 49315, Republic of Korea.
[2] Department of Chemistry, Kyung Hee University, 02453, Seoul, Republic of Korea.
* Correspondence: seojh@dau.ac.kr; walker@khu.ac.kr

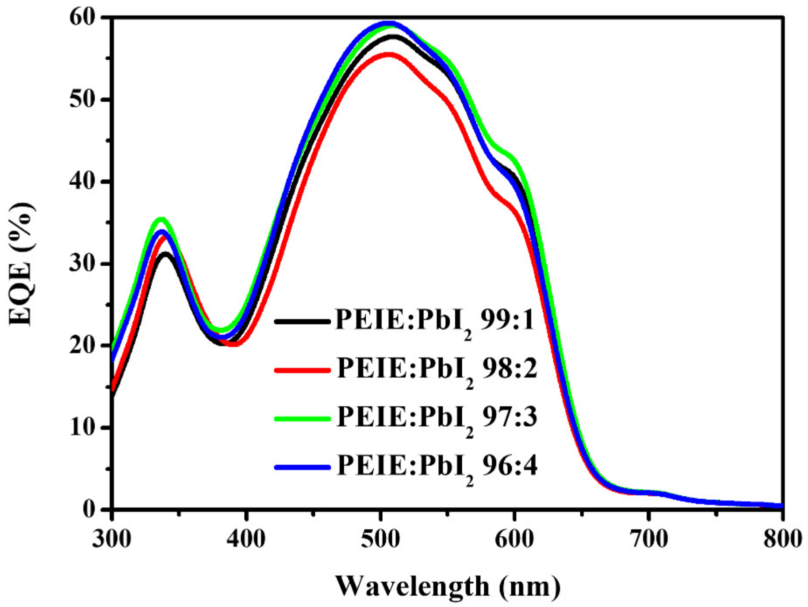

Figure S1. EQE characterization of inverted P3HT:$PC_{61}BM$ solar cells with various organic/inorganic hybrid composites.

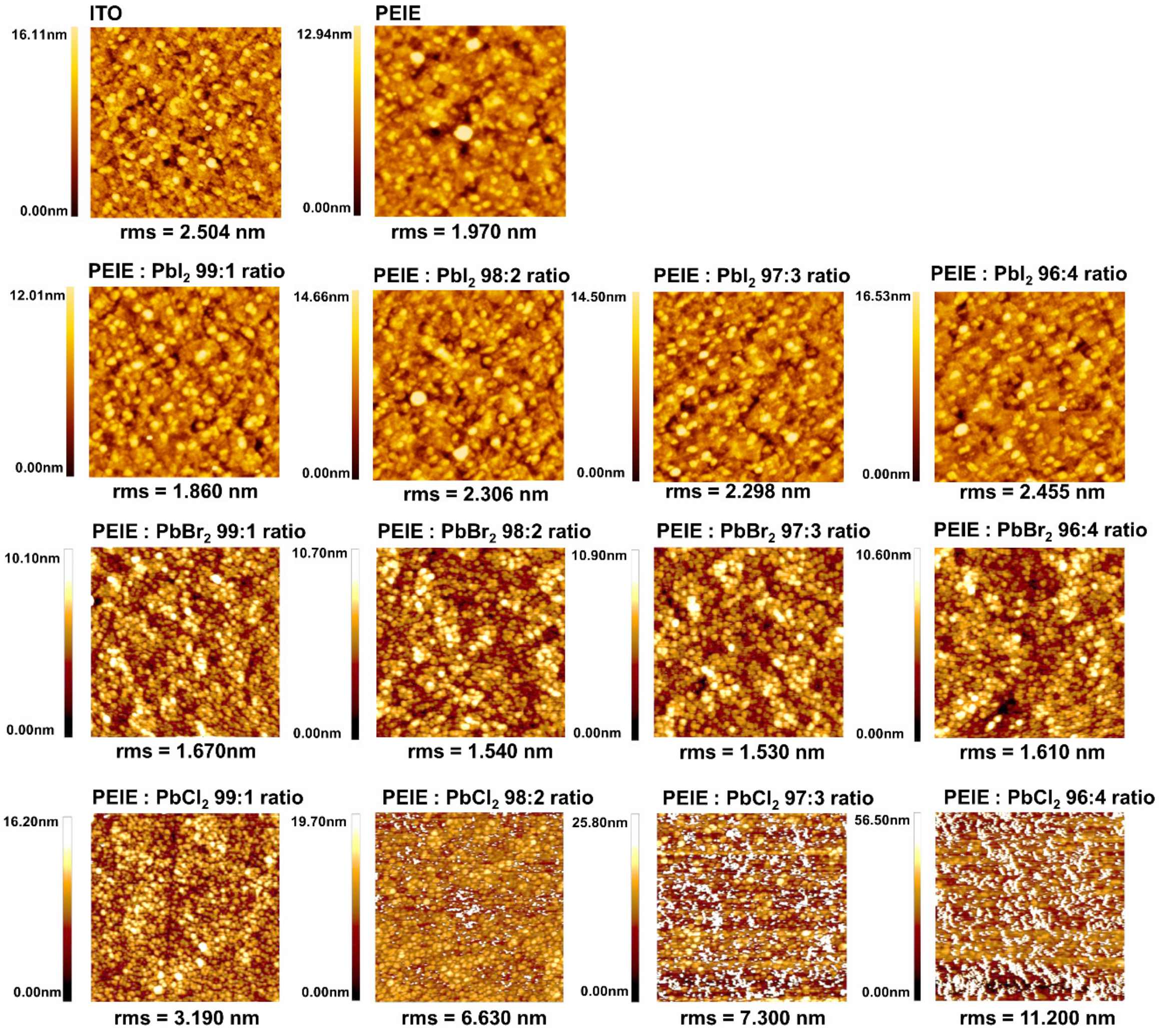

Figure S2. Surface topographic AFM images (size: 2 μm × 2 μm).

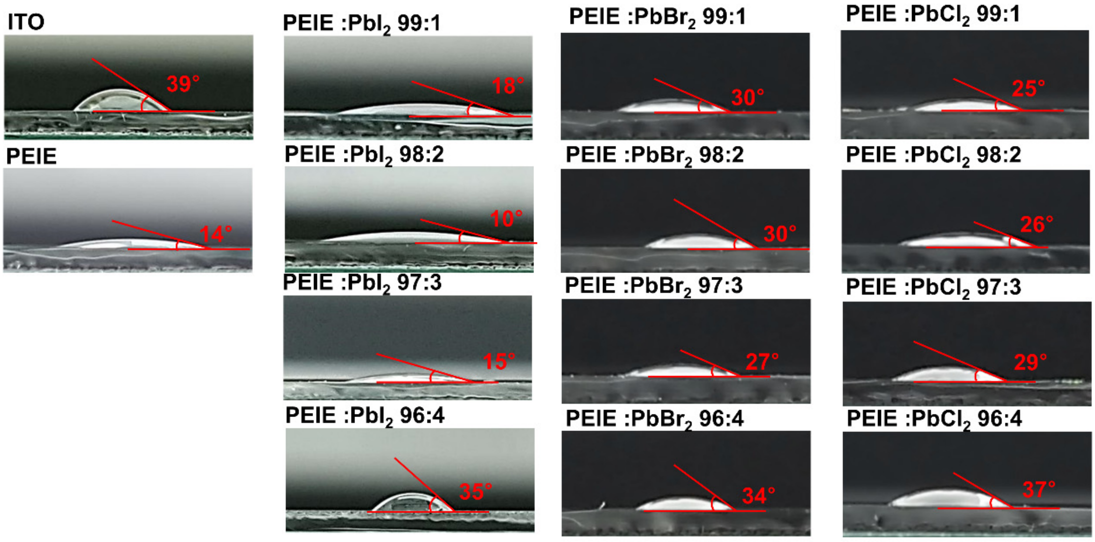

Figure S3. Photos of water droplets on different film surfaces.

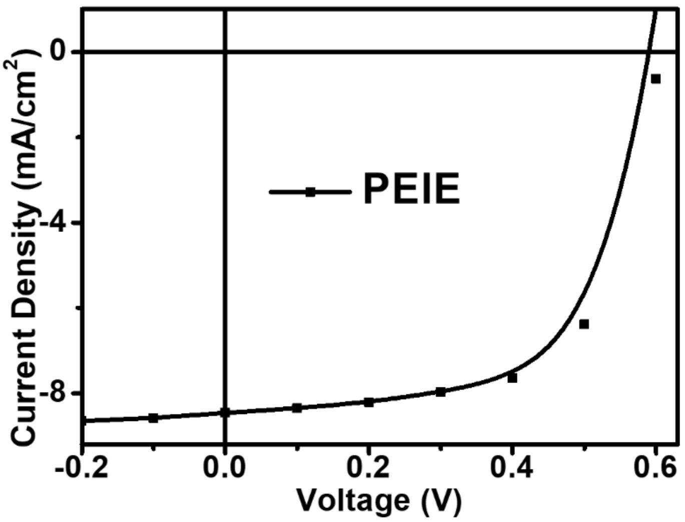

Figure S4. J-V characteristic of the inverted P3BT:$PC_{61}BM$ solar cells with a PEIE ETL under illumination.

Table S1. Conductivities of the ITO, PEIE and PEIE:PbX_2 films.

	Conductivity [S/sq]
ITO	0.1039 ± 1.91%
PEIE	0.1046 ± 1.83%
PEIE : $PbCl_2$ 99:1	0.1045 ± 1.48%
PEIE : $PbCl_2$ 98:2	0.1045 ± 1.52%
PEIE : $PbCl_2$ 97:3	0.1043 ± 1.59%
PEIE : $PbCl_2$ 96:4	0.1037 ± 1.60%
PEIE : $PbBr_2$ 99:1	0.1046 ± 1.73%
PEIE : $PbBr_2$ 98:2	0.1048 ± 1.13%
PEIE : $PbBr_2$ 97:3	0.1043 ± 1.52%
PEIE : $PbBr_2$ 96:4	0.1029 ± 1.53%
PEIE : PbI_2 99:1	0.1051 ± 0.98%
PEIE : PbI_2 98:2	0.1049 ± 0.55%
PEIE : PbI_2 97:3	0.1026 ± 2.91%
PEIE : PbI_2 96:4	0.1043 ± 1.46%
